# A Multiethnic Asian Perspective of Presumed Consent for Organ Donation: A Population-Based Perception Study

**DOI:** 10.3389/fpubh.2021.712584

**Published:** 2021-10-05

**Authors:** Mark D. Muthiah, Melissa Sin Hui Chua, Konstadina Griva, Ivan Low, Wen Hui Lim, Cheng Han Ng, Jeff Y. F. Hwang, Jason C. H. Yap, Shridhar G. Iyer, Glenn K. Bonney, Vathsala Anantharaman, Daniel Q. Huang, Eunice Xiang-Xuan Tan, Guan-Huei Lee, Alfred W. C. Kow, Bee Choo Tai

**Affiliations:** ^1^Division of Gastroenterology and Hepatology, National University Health System, Singapore, Singapore; ^2^National University Centre for Organ Transplantation, National University Health System, Singapore, Singapore; ^3^Yong Loo Lin School of Medicine, National University of Singapore, Singapore, Singapore; ^4^Lee Kong Chian School of Medicine, Nanyang Technological University, Singapore, Singapore; ^5^Saw Swee Hock School of Public Health, National University of Singapore, Singapore, Singapore; ^6^Department of Hepatobiliary and Pancreatic Surgery, National University Health System, Singapore, Singapore; ^7^Division of Nephrology, National University Health System, Singapore, Singapore

**Keywords:** presumed consent, organ transplantation, attitude, knowledge, policy

## Abstract

**Background:** Organ shortage is still a world-wide problem, resulting in long waiting lists for kidney, liver, and heart transplant candidates across many transplant centers globally. This has resulted in the move toward presumed consent to increase deceased organ donation rates. However, there remains a paucity of literature on public attitude and barriers regarding the opt-out system, with existing studies limited to Western nations. Therefore, this study aimed to understand public sentiment and different barriers toward organ donation from the perspective of Singapore, a highly diverse and multiethnic Asian society.

**Methods:** A cross-sectional community semi-structured interview was conducted in a public housing estate in Singapore. Pilot test was undertaken before participants were interviewed face-to-face by trained personnel. All statistical evaluations were conducted using Stata. The χ^2^-test compared subgroups based on patient characteristics while multivariable logistic regression identified predictors of willingness to donate/ assent. Effect estimates were quantified using odds ratio (OR).

**Findings:** Out of 799 individuals, 85% were agreeable to organ donation after death and 81% were willing to assent to donations of family members' organs, which declined by 16% (*p* < 0.001) after a clinical scenario was presented. Demographic factors including ethnicity, education, marital, and employment status affected willingness to donate and assent. Knowledge correlated significantly with willingness to donate and assent. In particular, knowledge regarding brain death irreversibility had the strongest correlation (AOR 2.15; 95% CI 1.60–2.89).

**Conclusions:** Organ donation rates remain low albeit presumed consent legislation, due to patient-level barriers, including but not limited to knowledge gaps, cultural values, religious backgrounds, and emotional impact at relatives' death. To effectively boost donor rates, it is crucial for policy makers to invest in public education and improve transplant provisions and family protocols.

## Introduction

The shortage of organs for transplant is a major limitation faced by transplant programmes worldwide. To help boost organ donation rates, countries have resorted to the use of presumed consent which assumes that an individual is agreeable to donating their organs after death (1). While presumed consent is thought to increase transplant rates, its true impact on increasing organ donation rates is questionable (2). A recent study demonstrated that countries which had adopted presumed consent legislation yielded fewer living donors with no significant difference in deceased donors as compared to countries with an opt-in approach to organ donation (3).

The use of presumed consent has been adopted by many countries including England (4), Scotland (5) Chile, and Finland (6) in recent years. Legislature on presumed consent was first introduced in 1987 in Singapore with the kidney, liver, cornea, and heart being the four main organs that can be procured from deceased donors. Compared to Western nations where the rates of donation from presumed consent range from to 5.9 to 46.9 per million population (pmp) (7), Singapore remains the sole Asian country adopting an opt-out system where the number of deceased organ donors remains low at 7–9 pmp per year despite the low national opt-out rate of 2.0–3.0%. In 2015, there were 334 patients on the waiting list for kidney transplantation, 54 patients on the liver transplant waiting list and 23 patients awaiting heart transplantation (8). Given that living donor organ transplantation remains limited with only 32 living kidney donors and 19 living liver donors in 2017 (9), this has consequently resulted in an average waiting time of 9 years for kidney transplant and around 1–2 years for liver and heart transplanation (10). Similarly, actualization of organ procurement from presumed consent is comparatively lower in Singapore compared to the United States (11, 12). A major barrier faced by Asian transplant programmes is the difference in cultural and societal values and practices (13). For instance, the Confucian idea of keeping an intact body during funeral rites outweighs the importance of organ donation (14), while conservative religious values regarding the sanctity of the body and strong family-centered systems can further decrease donations (15).

Despite the critical importance of public support in the success of presumed consent models, there is a paucity of data in the literature regarding attitudes and barriers toward this model of organ donation. To date, previous studies are mostly limited to Western nations (2, 16), with most only recruiting specific populations such as medical students, or healthcare professionals, possibly resulting in selection bias. Therefore, this study aims to bridge a gap in the understanding of public sentiment and different barriers toward presumed consent from the perspective of a highly diverse, multiethnic Asian society, in which Singapore holds a unique position in.

## Methods

A cross-sectional community semi-structured interview was conducted in Singapore, a country that adopts a presumed consent policy for deceased donor organ donations. Data were collected *via* door-to-door interviews, over 1 week in February 2017. The study was approved by the National Healthcare Group Domain Specific Review Board (NHG DSRB).

### Participants

In 2017, Singapore had a population of 5.6 million, of which 4 million were Singaporeans or SPR. 3.2 million of the 4 million stayed in public housing (17). Within each public housing estate, there is a predetermined proportion of persons from each major ethnic group as part of the local ethnic integration policy (18). This ensures a distribution of various ethnic groups within each housing estate that is similar to the population distribution. All Singaporeans and permanent residents in Singapore above the age of 21 years who were staying in the public housing estate of Bishan were eligible to participate in this door-to-door interview *via* cluster sampling if they were contactable. The age restriction was decided in accordance with the Human Organ Transplant Act (HOTA) which only applies to Singapore Citizens and Permanent Residents 21 years old and above. Bishan, one of the 55 public housing estates in Singapore, comprises 62,600 residents and was purposively selected for this study. As part of a two-stage cluster sampling method, the estate was divided into nine clusters according to geographical locations. Each cluster had 25–30 blocks of public housing apartments. In each cluster, five blocks were chosen by simple random sampling. All household units in each selected block were approached to do our study.

Households were approached by door-to-door knocking or doorbell ringing. If there was no response, the household would be approached on two further separate occasions at different times of the day. Only after three non-responses would a household be classified as “unavailable.” One participant was purposively selected from each household unit. The recruitment strategy for every household unit was to introduce the study to the first person who met the inclusion criteria. For households that did not have any eligible individuals to participate in the interview at the time of initial contact, the interviewers scheduled a follow-up visit and returned at the agreed timing when eligible participants were available.

Residents who declined to participate after an explanation of the study were classified as “declined participation.” Participants who started the interview, but prematurely terminated the interview were classified as “participants discontinuing study.” The ages, sex, and ethnicities of the residents who declined participation and discontinued the study were documented in addition to their reasons for declining participation.

### Semi-structured Interview Design

The semi-structured interview design was adapted from a model of willingness to become a potential organ donor (19). The interview was modified to include barriers to organ donation highlighted in the literature, and edited by public health specialists, statisticians, and clinicians who work closely with transplant patients. A pilot test with 40 participants was undertaken in English, Mandarin, Malay, Tamil, and the Chinese dialects of Teochew and Hokkien. This was done to access feasibility before embarking on the interview and to ensure face validity of study procedures and survey items such as comprehensibility, clarity, and linguistic adaptation for languages other than English. It was also during the pilot testing that the interview terms for these languages were standardized for administration of the interview. For the administration of the interview in the actual study, the questions were translated into English and Mandarin. It was required to be conducted in English, Mandarin, Dialects and Malay. Although Tamil is one of the four main languages in Singapore, we found that all participants who spoke Tamil were effectively bilingual in both English and Tamil and responded to the interview in English.

The interview elicited information on the participants' basic demographics, their willingness to donate their own organs after death, their willingness to assent to the donation of their family members' organs, and their knowledge and views on the opt-out system. The ethnicities of the participants were classified according to their national identification documentations, which use nationally agreed guidelines. The participants' willingness to assent to donations of their family members' organs was recorded in the general context, as well as in the context of a specific clinical scenario. The case scenario was developed based on guidelines to represent a typical case of a deceased donation health encounter (20). Relevance and clarity of case details were determined based on review by the multidisciplinary study team and the pilot study participants. Prior attempts at blood donation were taken as a measure of the participants' altruism (21).

Further information was collected on the participants' knowledge on the opt-out system, the organ donation process, as well as on brain death. These questions were divided into: (i) knowledge regarding the current laws on presumed consent; (ii) knowledge on the general processes in transplantation; and (iii) knowledge on brain death and its certification. Finally, the participants were asked for their reasons in supporting or rejecting organ donation *via* open-ended questions, which were recorded without prompts or options provided. The questionnaire has been included in the Supplementary Material.

### Interview Administration

Each participant was interviewed face-to-face, and their responses recorded by the interviewers. The interviewers were trained by a single trainer prior to the study. Responses to further clarifications by interview participants were standardized based on commonly asked questions during the pilot study. The interview was conducted in English when possible. For participants who were unable to speak English, interviewers who were conversant in the preferred languages or dialects were sent back to the houses concerned to conduct the interview in those languages or dialects. Interviewers were trained in the language and dialect terms to be used before the study.

### Outcomes

The primary outcome measure was to measure the overall willingness to donate one's own organs. The secondary outcome measures were (1) willingness to assent to donation of family members' organs within a clinical scenario (2) the impact of knowledge gaps on willingness to assent to the donation of family members organs within a clinical scenario (3) reasons for or against organ donation.

### Sample Size Estimation and Statistical Analysis

We postulated that the proportion of assent was 85% with a margin of error of 2.5%. Using a precision-based approach and assuming a 95% level of confidence, a minimum sample size of 784 would be required. Further assuming a rejection rate of 2 in 3, a total of 2,352 participants needed to be recruited for the semi-structured interview.

The patient characteristics that were categorical in nature were summarized in terms of frequencies and percentages, and differences between subgroups based on willingness to donate or assent within a given scenario were compared using the χ^2^-test. Variables that were significant at the 5% level in these comparisons were further considered for inclusion in the multivariable logistic regression to identify predictors of willingness to donate and willingness to assent within a clinical scenario. Furthermore, the difference in proportions that were willing to donate and willing to assent within a clinical scenario was compared using McNemar's test for paired proportion. The effect estimates were quantified using odds ratio (OR) and its associated 95% confidence. The association between willingness to donate and willingness to assent within a clinical scenario with the number of questions answered correctly were also assessed using the χ^2^-test. All statistical evaluations were conducted using Stata version 16, assuming a two-sided test at the 5% level of significance.

### Role of the Funding Source

The funder of the study had no role in study design, data collection, data analysis, data interpretation, or writing of the paper. The corresponding author had full access to all the data in the study and all authors shared final responsibility for the decision to submit for publication.

## Results

**Figure 1:**
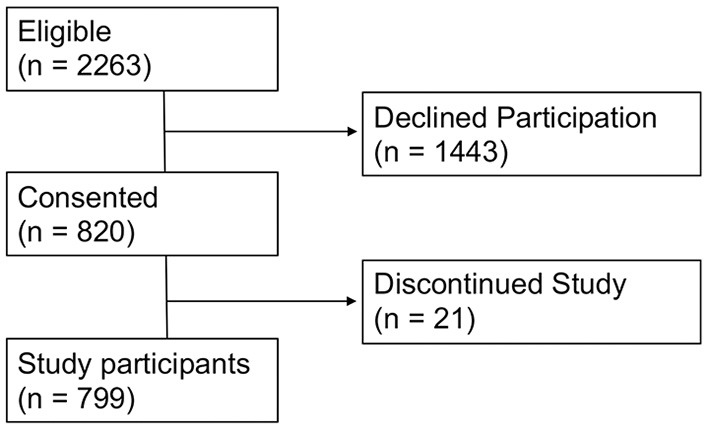
Overall flow of the study.

Two-thousand two hundred and sixty-three households were eligible to participate from the nine clusters. Out of 2,263 household units in all selected blocks from the nine clusters, residents from 820 units consented to participating in the study, while residents from 1,443 units declined participation. Of the 820 residents who initially agreed to be interviewed, 21 discontinued the study. The analysis of this study included only the 799 participants who completed the interview (Figure 1). Data on the residents who declined participation or discontinued the study have been included (Supplementary Table 1).

### Baseline Demographics

Of the 799 participants completing the study, 58% were female and the majority (42%) were in the 41–60 years age group. The participants were predominantly Chinese (79%) with 13% Indians and 7% Malays, largely reflecting the ethnic composition in Singapore. In terms of religious affiliations, they were primarily Christians (32%), Buddhists (25%), or atheists (24%). A detailed description of the demographics of the study population is shown in Table 1.

**Table 1:** Demographic characteristics of the participants by their willingness to donate.

**Characteristics**	**Number of participants willing to donate (%)**		***p*-Value**
	**Yes**	**No**	
	**(*n* = 679)**	**(*n* = 120)**	
*Age (years)*			0.004^*^
21–40	209 (30.8)	23 (19.2)	
41–60	288 (42.4)	49 (40.8)	
>60	182 (26.8)	48 (40.0)	
*Sex*			0.195
Male	292 (43.0)	44 (36.7)	
Female	387 (57.0)	76 (63.3)	
*Ethnicity*			0.001^*^
Chinese	542 (79.8)	88 (73.3)	
Malay	38 (5.6)	18 (15.0)	
Indian	92 (13.6)	11 (9.2)	
Others	7 (1.0)	3 (2.5)	
*Religion*			0.015^*^
Atheist	169 (24.9)	23 (19.2)	
Buddhist	160 (23.6)	38 (31.7)	
Christian	226 (33.3)	29 (24.2)	
Muslim	51 (7.5)	18 (15.0)	
Hindu	53 (7.8)	6 (5.0)	
Taoist	17 (2.5)	5 (4.2)	
Other	3 (0.4)	1 (0.8)	
*Marital status*			0.183
Single	171 (25.2)	24 (20.0)	
Married	481 (70.8)	87 (72.5)	
Divorced	13 (1.9)	3 (2.5)	
Widowed	14 (2.1)	6 (5.0)	
*Employment*			0.025^*^
Full-time	340 (50.1)	44 (36.7)	
Part-time	77 (11.3)	18 (15.0)	
Not working	262 (38.6)	58 (48.3)	
*Education*			<0.001^*^
Secondary and below	214 (31.5)	71 (59.2)	
Pre-university/polytechnic	200 (29.5)	27 (22.5)	
University	265 (39.0)	22 (18.3)	
*Attempted to donate blood*			<0.001^*^
Yes	341 (50.2)	35 (29.2)	
No	338 (49.8)	85 (70.8)	
*Willing to be a living donor*			<0.001^*^
Yes	645 (95.0)	92 (76.7)	
No	34 (5.0)	28 (23.3)	
*Willing to receive an organ from a living donor*			<0.001^*^
Yes	505 (74.4)	50 (41.7)	
No	174 (25.6)	70 (58.3)	

* *P-value < 0.05 is statistically significant*.

### Willingness to Donate One's Own Organs After Death

Of the 799 participants, 85% (*n* = 679, 95% CI 82–87) were willing to donate their organs after brain death. In the bivariate association with willingness to donate, the following variables were significant at the 5% level: age, ethnicity, religion, employment status, education, attempted to donate blood, willingness to be a living donor, and willingness to receive an organ from a living donor.

A multivariable logistic regression analysis demonstrated that ethnicity, level of education, attempts to donate blood, and willingness to be living organ donors were independently associated with willingness to donate. Compared to Chinese participants, Malay participants were less willing to donate their organs after death (adjusted OR 0.33; 95% CI 0.17–0.62). Similarly, compared to participants with secondary level education and below, participants with non-degree tertiary level education, and University education were more willing to donate their organs after death (AOR 1.89; 95% CI 1.14–3.15 and 3.32; 95% CI 1.92–5.75, respectively). Participants who had attempted to donate blood (AOR 1.80, 95% CI 1.15–2.82), or who were willing to be living organ donors (AOR 4.63, 95% CI 2.58–8.31) were also more likely to be willing to donate their organs after death (Table 2).

**Table 2:** Significant predictors of being willing to donate in a multivariable logistic regression.

**Characteristics**	**OR**	**95% CI**	***p*-value**
*Ethnicity*			0.002^*^
Chinese	Ref	–	
Malay	0.33	0.17–0.62	0.001^*^
Indian	0.98	0.49–1.97	0.962
Others	0.23	0.05–1.01	0.051
*Education*			<0.001^*^
Secondary and below	Ref	–	
Pre-university/polytechnic	1.89	1.14–3.15	0.014^*^
University	3.32	1.92–5.75	<0.001^*^
Attempted to donate blood	1.80	1.15–2.82	<0.001^*^
Willing to be a living donor	4.63	2.58–8.31	<0.001^*^

* *P-value < 0.05 is statistically significant*.

### Willingness to Assent to Family Members' Organs Being Donated Declines in the Context of a Scenario

Of the 799 participants, 81% (*n* = 649, 95% CI 78–84) were willing to assent to the donation of their family members' organs after brain death. However, when presented with a realistic scenario, only 65% (*n* = 521, 95% CI 62–69) were willing to assent to the donation of their family members' organs after brain death. The difference in paired proportion between willingness to assent without a clinical scenario and willingness to assent after a clinical scenario was presented was 16% (95% CI 13–19, *p* < 0.001).

### Factors Associated With Willingness to Assent to Family Members' Organs Being Donated in the Context of a Clinical Scenario

In the bivariate analysis, variables that were significantly associated with willingness to assent at the 5% level included age, ethnicity, religion, marital status employment status, attempt to donate blood, and willingness to be a living donor (Supplementary Table 2). A multivariable logistic regression analysis demonstrated that age, ethnicity, marital status, and employment status were associated with willingness to assent after being presented a clinical scenario (Table 3). Compared to Chinese, Indians were more likely to be willing to assent (AOR 1.72; 95% CI 1.05–2.81). Compared to single participants, married and widowed individuals in particular were less likely to be willing to assent (AOR 0.65; 95% CI 0.43–0.96, and 0.20; 95% CI 0.07–0.57 respectively).

**Table 3:** Significant predictors of being willing to assent in a clinical scenario in a multivariable logistic regression.

**Characteristics**	**OR**	**95% CI**	***P*-value**
*Age (years)*			0.004^*^
21–40	Ref	–	
41–60	0.79	0.53–1.16	0.226
>60	1.59	0.98–2.59	0.063
*Ethnicity*			0.040^*^
Chinese	Ref	–	
Malay	0.69	0.39–1.22	0.206
Indian	1.72	1.05–2.81	0.032^*^
Others	2.73	0.55–13.54	0.218
*Marital status*			0.009^*^
Single	Ref	–	
Married	0.65	0.43–0.96	0.033^*^
Divorced	0.40	0.13–1.18	0.096
Widowed	0.20	0.07–0.57	0.002^*^
*Employment*			0.043^*^
Full-time	Ref	–	
Part-time	0.79	0.49–1.27	0.329
Not working	1.41	0.98–2.02	0.064
Attempted to donate blood	1.43	1.05–1.95	0.024^*^
Willing to be a living donor	1.92	1.10–3.34	0.022^*^

* *P-value < 0.05 is statistically significant*.

### Knowledge Gaps Affect Willingness to Donate and Willingness to Assent

Out of the 14 knowledge questions, the most common misconception was that brain death certification was not a stringent process (11.5% correct), followed by knowing the four organs covered under the HOTA (12.9% correct). Correct answers on questions concerning brain death testing had the highest association with being willing to assent to the donation of family members' organs. Knowing that brain death is irreversible and knowing that organs could be procured from brain dead patients were the strongest predictors of willingness to assent (OR 2.15; 95% CI 1.60–2.89 and 2.08; 95% CI 1.50–2.89, respectively). Being aware that HOTA was an opt-out system (based on presumed consent) was the strongest predictor of willingness to donate (OR 3.41; 95% CI 2.08–5.59). The details of the questions answered have been summarized in Table 4 and Supplementary Table 3.

**Table 4:** Knowledge questions and their association with willingness to assent.

**Question theme**	**Domain**	**Number of participants answering correctly (%)**	**Willingness to assent in a clinical scenario**	
			**Odds ratio (95% CI)**	***P*-value**
Knows four organs covered by HOTA	HOTA Law	103 (12.9)	1.42 (0.90–2.24)	0.130
Aware of the HOTA being an opt out system	HOTA Law	306 (38.3)	1.55 (1.14–2.11)	0.005^*^
Knows the HOTA covers for those aged ≥21 years old	HOTA Law	323 (40.4)	1.27 (0.94–1.71)	0.116
Knows what the organs under the HOTA is used for	HOTA Law	363 (45.4)	0.90 (0.67–1.21)	0.483
Understands concepts of fair organ allocation	HOTA Law	483 (60.5)	1.51 (1.12–2.03)	0.006^*^
Aware of a local act governing organ donation	HOTA Law	529 (66.2)	1.28 (0.94–1.73)	0.114
Aware that procured organs are unlikely to pass disease to recipients after stringent screening	Transplant processes	248 (31.0)	1.72 (1.24–2.40)	0.001^*^
Aware that the government pays for hospitalization under the HOTA implementation	Transplant processes	341 (42.7)	1.33 (0.99–1.79)	0.058
Aware that organs procured from deceased donors are a viable alternative to living donors organ transplant to potentially extend survival for transplant candidates	Transplant processes	436 (54.6)	1.62 (1.21–2.17)	0.001^*^
Knows that a deceased relative's body is returned to the family after an organ transplant	Transplant processes	561 (70.2)	1.37 (1.00–1.88)	0.048^*^
Knows that organ transplantation is generally a last resort	Transplant processes	625 (78.2)	1.39 (0.99–1.97)	0.060
Aware of the stringent brain death certification process	Brain death	92 (11.5)	1.33 (0.82–2.13)	0.244
Knows brain death is irreversible	Brain death	429 (53.7)	2.15 (1.60–2.89)	<0.001^*^
Knows organs can be procured from brain dead patients	Brain death	600 (75.1)	2.08 (1.50–2.89)	<0.001^*^

* *P-value < 0.05 is statistically significant, HOTA, Human Organ Transplant Act*.

### Knowledge Enhances Willingness to Donate and Willingness to Assent

**Figure 2:**
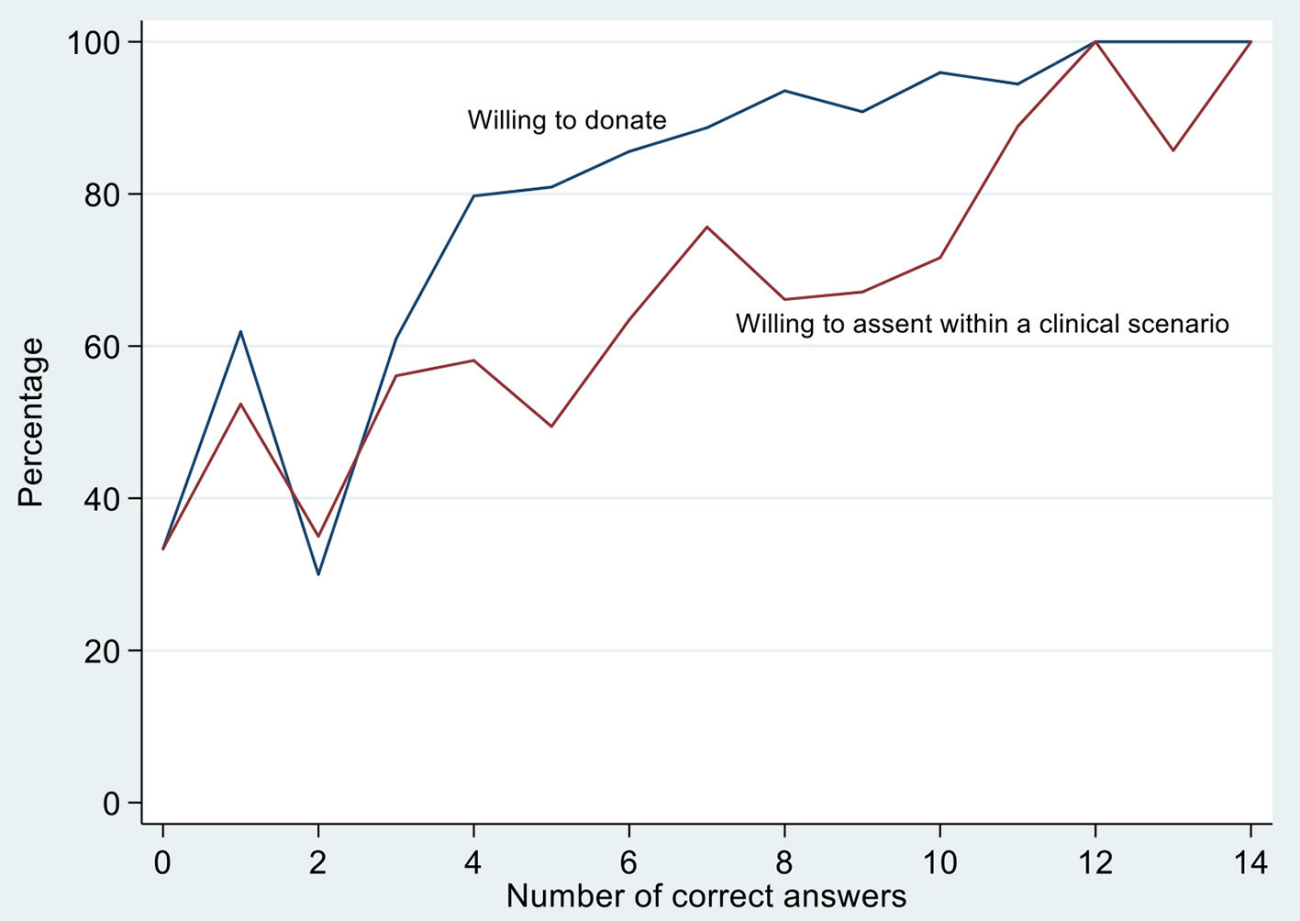
Association between the number of questions answered correctly and willingness to donate and assent to donation of family members' organs. *In this figure, answering more questions correctly is significantly associated with willingness to donate (blue) and willingness to assent to donation of a family members organs after being presented with a clinical scenario (red). *P*-values are both < 0.001 based on the chi-squared test.

There was a significant positive association between the number of questions answered correctly and willingness to donate (*p* < 0.001). This was also seen between the number of questions answered correctly and willingness to assent in the clinical scenario (*p* < 0.001) (Figure 2).

### Reasons in Support of and Against Organ Donation

When asked, the most cited reason in support of organ donation among the respondents was “to do good,” followed by “organs should not go to waste.” Only 6.3% of the respondents felt that the fact that their relative did not opt-out of HOTA suggested he/she was agreeable to organ donation. The most commonly cited reasons against organ donation were “too upset and unprepared to make a decision” and “want the body to be intact for religious reasons.” The top barriers against organ donation identified were emotional impact at the time of a loved one's passing, as well as cultural or religious reasons. These have been summarized in Tables 5, 6. Other open text responses have been aggregated into categories based on their themes, and listed in Supplementary Tables 4A–C.

**Table 5:** Reasons for supporting organ donation.

**Reasons supporting organ donation**	**Number of participants who cited this reason (%)**
To do good	641 (80.2)
My deceased relative is already dead and has no need for his/her organs, which should not go to waste	318 (39.8)
My deceased relative previously expressed the intention to donate his/her organs after death, and I want to respect his/her wishes	56 (7.0)
My deceased relative did not opt out of the HOTA so I assume he/she is agreeable to organ donation after death	50 (6.3)
My deceased relative can continue to live through others	46 (5.6)
I want to contribute the organs for research and medical use	13 (1.6)
My religion and beliefs support organ donation	11 (1.4)
Prepared to let the family member go	10 (1.3)
Had experiences regarding myself or loved ones regarding organ donation	7 (0.9)
Good education and awareness regarding organ donation	5 (0.6)

*HOTA, Human Organ Transplant Act*.

**Table 6:** Reasons against supporting organ donation.

**Reason against supporting organ donation**	**Number of participants who cited this reason (%)**
I am too emotionally involved with the recipient and will be too upset and unprepared to make a decision	322 (40.3)
I want my deceased relative to be bodily intact after death due to religion/superstition	159 (19.9)
My relative may not have passed away yet or my relative may still be alive	123 (15.4)
My deceased relative did not actively agree to this, and my relative may not have pondered over it properly before dying	101 (12.6)
There is insufficient time given for grieving before organs are procured	92 (11.5)
The brain death certification could be inaccurate	70 (8.8)
I do not fully understand or trust the organ transplantation process	32 (4.0)
Other family members must be consulted before a decision can be made	32 (4.0)
I do not want my relative to go through additional procedures involved in organ procurement after all he/she has been through	28 (2.5)
It disturbs the natural proceedings (e.g., funeral or burial) after the passing of a relative as the body is held in the ICU until the organs are procured	25 (3.1)
The doctors would stop putting in their best effort to save my relative because they want to procure his/her organs	12 (1.5)
Ill effects to the recipient	12 (1.5)
The HOTA should be opt in or not opt out, as the deceased may not have been aware	9 (1.1)
All responses due to misconceptions about the HOTA	7 (0.9)
I am not sure if the organs are going to a good deserving person or someone I know	7 (0.9)
Medical professionals are not tactful and empathetic enough	2 (0.3)

*HOTA, Human Organ Transplant Act*.

## Discussion

The current global shortage of organs has resulted in the move toward presumed consent. While the efficacy and ethics of presumed consent remains debatable (22), certain countries including Chile and Austria have unequivocally increased donation rates successfully through this model (23) (Supplementary Material 6). To date, Singapore remains the only country in Asia with an opt-out system although other Asian countries have discussed the possibility of legislating presumed consent (13). With the stark disparity between the cultural and societal values of Caucasians and Asians, this article presents novel quantitative and qualitative findings regarding attitudinal and knowledge barriers that impede locals' willingness to donate and assent and thus, addresses a key gap in the current understanding of presumed consent from an Asian perspective.

Despite the low national opt-out rates in Singapore's presumed consent system (2–3%), this study revealed a large discrepancy with up to 15% of individuals refusing to donate their organs after death. Importantly, the main barriers against organ donation were emotional impact at the time of a loved one's passing, as well as cultural or religious reasons. Acute psychological stress, shock, and disbelief among family members of deceased donors have been shown to affect the decision-making matrix to assent to organ donation (24). These findings underscore the importance of giving families time to adjust to the shock of the death before considering donation. This may be facilitated through decoupling the pronouncement of death from the request for organ donation and increasing family support during grieving to protect psychological well-being of family members while increasing the likelihood of assent (25, 26). Comparatively, cultural barriers, namely the concept of maintaining an intact body shaped by Confucian-principles of filial piety has been shown to discourage organ donations (27, 28) which remain a major issue amidst a historically conservative geographical region. Our results also showed that other factors such as disparities among different groups, in terms of age, ethnicity, marital status, employment status, and education level affected willingness to donate and willingness to assent. Notwithstanding, attempts to donate blood was found to be a potential marker of altruism when considering organ donation, similar to Narayanan et al. (21).

Notably, this study demonstrated a clear correlation between knowledge gaps and decreased willingness to donate and willingness to assent to the donation of family members' organs (Figure 2). Surprisingly, despite presumed consent being legislated in Singapore since 1983, only 38.3% of participants knew that HOTA is an opt-out system which was the strongest predictor of being willing to donate one's own organs after death. These findings have clear policy implications as it suggests that the mere implementation of a presumed consent system is insufficient to boost organ availability (29). Extensive public education to disseminate knowledge on the presumed consent system, benefits of organ donation, transplant process, and brain death criteria thus remains the key in normalizing organ donation, as supported by study participants.

Importantly, our study demonstrated that 85% of the population are willing to donate after brain death. While the high rates of willingness to donate appear comforting, previous studies have reported that east Asians demonstrate a higher social desirability bias as compared to Americans (30, 31). We hence postulate that high social desirability bias among Singaporean participants may have inflated reported willingness to donate, whereas actual donation rates remain low in Singapore.

### Implications on Practice

Although legislative environment plays a role to incentivize organ donation (32), previous studies have demonstrated that family refusal can impede organ donation (33, 34). With the significantly high dissent rate of 35% toward donation of family members' organ in our study comparable to other countries (3), strategies to improve communication with next-of-kin are crucial to the success of presumed consent models. For instance, training programs including the European Donor Hospital Education Programme (35) and the Donor Action Program (36) are designed to help improve transplant staff's language use and dialogue skills regarding organ donation which can influence relatives' decision to assent. Currently, most programs stem from Europe (37) in stark comparison to Asia where there is a lack of such measures (38), with the exception of the 2019 Organ Donation Innovative Strategies for Southeast Asia program (39). Considering that familism and filial piety reign strong as cultural features of Asian societies as compared to Western societies (40, 41), provision of family-based organ donation services to facilitate early familial discussions about donation intentions should be considered in Asian nations. Spain, the country with the highest organ donation rates in the world, provides a good role model in the successful development of family protocols, where intensive care physicians set dedicated time aside to coordinate key roles in organ donation and conduct discussions with family members (42). Other complementary policies such as encouraging individuals to document their preferences in a registry may also help reduce family ambiguity and ambivalence with regards to deceased donors' wishes as discussed in previous literature (43, 44).

Another critical issue to be tackled is the knowledge deficit regarding organ donation, particularly around the opt-out legislature and concept of brain death. Public awareness initiatives *via* media campaigns alongside active partnerships with journalists and phased education have been shown to address misconceptions and bridge the disconnect between attitudes and willingness to donate (45, 46). Novel behavioral intervention strategies can further complement these initiatives through indirect suggestion and positive reinforcement to help bypass underlying cognitive obstacles (47). Indeed, investment into public education and family involvement are key elements for the success of presumed consent models. This study thus serves as a call to action for policy makers to address public attitudes to boost organ donation rates under the opt-out approach.

### Strengths and Limitations

The strength of this study is that it is a large population-based multiethnic study assessing perceptions on organ donation, which was conducted in a country that has 30 years of experience with presumed consent legislation. However, there are several limitations to the study. As with most perception-based studies, the intention to donate is not equivalent to donation. Additionally, this study also uses a hypothetical scenario to assess participants' willingness to assent to donation of a family members' organs and demonstrated a significant reduction in the willingness of family members to assent to donation of a patients' organs after a specific clinical scenario was given to them. While it has not been used in similar studies before, the case scenario is an established research design widely used across various disciplines to generate in-depth understanding of the issue of enquiry in a real-life context. However, there remains no established nationwide statistic on the family refusal rate with regards to deceased organ donation which warrants further investigation in the future. Other limitations include the limited size of some population subgroups and the high (65%) rejection rate of the study. Previous studies have demonstrated that there is not a direct correlation between response rate and validity (48). The predicted response rate was also accounted for in the calculation of recruitment target to achieve a minimum sample size of 784 responders, validating the results of this study. Furthermore, the response rate is comparable to other similar studies as reported in Supplementary Table 6. Ideally, the interview would have been piloted with 10% of the minimum sample size but the pilot sample size was limited to 40 patients given recruitment constraints.

### Conclusion

In conclusion, there is a stark contrast between patients presumed to consent to organ donation, and the actual proportion of patients agreeable to organ donation in Singapore. Besides sociodemographic factors, the main barriers to organ donation include knowledge gaps, sociocultural, and religious backgrounds, as well as emotional impact at time of death of relative. While the presumed consent system holds much potential to increase organ donation rates, this study provides clear evidence that policy makers need to invest heavily in increasing transplant provisions, while promoting public education on multiple aspects of organ donation to effectively boost donor rates. The process of obtaining assent from family members to procure organs from deceased relatives should also be further refined, centered around empathy, and sensitivity.

## Data Availability Statement

The raw data supporting the conclusions of this article will be made available by the authors, without undue reservation.

## Ethics Statement

The studies involving human participants were reviewed and approved by National Healthcare Group Domain Specific Review Board. The patients/participants provided their written informed consent to participate in this study.

## Author Contributions

MM, MC, KG, IL, JH, and JY contributed to the acquisition of data, analysis and interpretation of data, and drafting of the article. SI, GB, VA, WL, CN, DH, ET, G-HL, AK, and BT aided in revising the article critically for important intellectual content. All authors read and gave final approval of the version to be submitted.

## Funding

DH declares research support from Exxon Mobil-NUS Research Fellowship for Clinicians. The funder of the study had no role in study design, data collection, data analysis, data interpretation, or writing of the paper.

## Conflict of Interest

The authors declare that the research was conducted in the absence of any commercial or financial relationships that could be construed as a potential conflict of interest.

## Publisher's Note

All claims expressed in this article are solely those of the authors and do not necessarily represent those of their affiliated organizations, or those of the publisher, the editors and the reviewers. Any product that may be evaluated in this article, or claim that may be made by its manufacturer, is not guaranteed or endorsed by the publisher.

## Abbreviations

HOTA: Human Organ Transplant Act
PMP: per million population.
