# Supplementary material for: A Multiethnic Asian Perspective of Presumed Consent for Organ Donation: A Population-Based Perception Study
Source: Front Public Health. 2021 Oct 5;9:712584. doi: 10.3389/fpubh.2021.712584 (PMC8525798; doi:10.3389/fpubh.2021.712584)
Supplement: Supplementary file 1 [file Table_1.DOCX]

**Supplementary Table 1: Demographic characteristics of the participants and the residents who declined participation or discontinued the study**

| **Characteristics** | **Participants**  **(*n* = 799)** | **Residents declining participation / discontinuing study**  **(*n* = 1464)** | ***p*-value** |
| --- | --- | --- | --- |
| *Age (year)* (%)  21 – 40   1. – 60   > 60 | 232 (29.0)  337 (42.2)  230 (28.8) | 362 (24.7)  640 (43.7)  462 (31.6) | 0.073 |
| *Gender* (%)  Male  Female | 336 ((42.1)  463 (58.0) | 594 (40.6)  870 (59.4) | 0.494 |
| *Ethnicity* (%)  Chinese  Malay  Indian  Others | 630 (78.9)  56 (7.0)  103 (12.9)  10 (1.3) | 1257 (85.9)  75 (5.12)  113 (7.12)  19 (1.30) | < 0.001 |

**Supplementary Table 2: Demographic characteristics of willingness to assent within a clinical scenario**

| **Characteristics** | **Willing to assent within a clinical scenario** | | **p-value** |
| --- | --- | --- | --- |
|  | **Yes**  **(n = 521)** | **No**  **(n = 278)** |  |
| *Age (year)*  21 – 40   1. – 60   > 60 | 160 (30.7)  195 (37.4)  166 (31.9) | 72 (25.9)  142 (51.1)  64 (23.0) | 0.001 |
| *Gender*  Male  Female | 229 (44.0)  292 (56.1) | 107 (38.5)  171 (61.5) | 0.136 |
| *Ethnicity*  Chinese  Malay  Indian  Others | 407 (78.1)  28 (5.4)  78 (15.0)  8 (1.5) | 223 (80.2)  28 (10.1)  25 (9.0)  2 (0.7) | 0.008 |
| *Religion*  Atheist  Buddhist  Christian  Muslim  Hindu  Taoist  Other | 131 (25.1)  115 (22.1)  173 (33.2)  39 (7.5)  47 (9.0)  14 (2.7)  2 (0.4) | 61 (21.9)  83 (29.9)  82 (29.5)  30 (10.8)  12 (4.3)  8 (2.9)  2 (0.7) | 0.028 |
| *Marital status*  Single  Married  Divorced  Widowed | 141 (27.1)  363 (69.7)  9 (1.7)  8 (1.5) | 54 (19.4)  205 (73.7)  7 (2.5)  12 (4.3) | 0.012 |
| *Employment*  Full-time  Part-time  Not working | 240 (46.1)  53 (10.2)  228 (47.8) | 144 (51.8)  42 (15.1)  92 (33.1) | 0.006 |
| *Education*  Secondary and below  Pre-University/Polytechnic  University | 174 (33.4)  152 (29.2)  195 (37.4) | 111 (39.9)  75 (27.0)  92 (33.1) | 0.181 |
| *Attempted to donate blood*  Yes  No | 261 (50.1)  260 (49.9) | 115 (41.4)  163 (58.6) | 0.019 |
| *Willing to be a living donor*  Yes  No | 488 (93.7)  33 (6.3) | 249 (89.6)  29 (10.4) | 0.039 |
| *Willing to receive an organ from a living donor*  Yes  No | 384 (73.7)  137 (26.3) | 191 (68.7)  87 (31.3) | 0.134 |

**Supplementary Table 3: Knowledge questions and the correlation with being willing to donate one’s own organs after death**

| **Question theme** | **Domain** | **Number of participants answering correctly (%)** | **Willingness to donate one’s own organs after death** | |
| --- | --- | --- | --- | --- |
|  |  |  | **Odds Ratio (95% CI)** | **P Value** |
| Knows 4 organs covered by HOTA | HOTA Law | 103 (12.9) | 1.98 (0.97 **–** 4.04) | 0.056 |
| Aware of HOTA being an opt out system | HOTA Law | 306 (38.3) | **3.41 (2.08 – 5.59)** | **< 0.001** |
| Knew HOTA covers for those aged ≥ 21 years old | HOTA Law | 323 (40.4) | 2.28 (1.47 **–** 3.54) | < 0.001 |
| Knew what the organs under HOTA is used for | HOTA Law | 363 (45.4) | 1.25 (0.84 **–** 1.85) | 0.272 |
| Understands concepts of fair organ allocation | HOTA Law | 483 (60.5) | **3.03 (2.03 – 4.53)** | **< 0.001** |
| Aware of a local act governing organ donation | HOTA Law | 529 (66.2) | 2.23 (1.51 **–** 3.31) | < 0.001 |
| Aware that procured organs are unlikely to pass disease to recipient after stringent screening | Transplant processes | 248 (31.0) | **2.37 (1.44 – 3.89)** | **< 0.001** |
| Aware that the government pays for hospitalization under HOTA implementation | Transplant processes | 341 (42.7) | 1.90 (1.25 **–** 2.89) | 0.002 |
| Aware that organs procured from deceased donors are a viable alternative to living donor organs to potentially extend survival for transplant candidates | Transplant processes | 436 (54.6) | **2.80 (1.86 – 4.21)** | **< 0.001** |
| Knows that deceased relative’s bodies are returned to the family after organ transplant | Transplant processes | 561 (70.2) | **2.20 (1.48 – 3.28)** | **< 0.001** |
| Knows organ transplantation is generally a last resort | Transplant processes | 625 (78.2) | 1.60 (1.03 **–** 2.47) | 0.033 |
| Aware of stringent brain death certification process | Brain death | 92 (11.5) | 2.31 (1.04 **–** 5.12) | 0.034 |
| Knows brain death is irreversible | Brain death | 429 (53.7) | **1.70 (1.15 – 2.52)** | **0.008** |
| Knows organs can be procured from brain dead patients | Brain death | 600 (75.1) | **2.65 (1.77 – 3.98)** | **<0.001** |

**Supplementary Table 4: Responses to open ended questions**

**Supplementary Table 4A: Description of state of emotion after listening to the clinical scenario (Question 30)**

| **Explanation** | **N (%)** |
| --- | --- |
| Accepting, feeling that a part of their relative can live on | 394 (49.3) |
| Devastated | 49 (6.1) |
| Angry | 70 (8.8) |
| Uncertain about donation as have not thought about this before | 68 (8.5) |
| At a loss about donation and would need help with decision making | 193 (24.1) |
| Doubtful about donation as R may still be alive | 79 (9.9) |
| Accepting because R did not opt out of HOTA | 25 (3.1) |
| R did not explicitly agree to HOTA | 27 (3.4) |
| Overwhelmed | 55 (6.9) |
| Sad, shocked | 61 (7.6) |
| Overwhelmed, angry, uncertain, sad, shocked | 374 (46.8) |
| Only acceptance | 317 (39.7) |

**Supplementary Table 4B: Open text responses justifying decision to assent / object to R’s organ donation in the clinical scenario (Question 32)**

| **Explanation** | **N (%)** |
| --- | --- |
| R’s donation would save life | 298 (37.3) |
| R’s donation would allow him to live on | 12 (1.5) |
| R’s donation allows the organs be utilised and not wasted | 119 (14.9) |
| R’s donation follows the fact that he did not opt out | 118 (14.8) |
| Not comfortable to assent to R’s donation as R may not be dead | 88 (11.0) |
| Not comfortable to assent to R’s donation as it was not right to take organs | 22 (2.8) |
| Not comfortable to assent to R’s donation as the body needs to be whole | 46 (5.8) |
| Not comfortable to assent to R’s donation as there was insufficient time for grieving | 62 (7.8) |
| Not comfortable to assent to R’s donation as the body would become unpleasant after organ donation | 1 (0.1) |
| Not comfortable to assent to R’s donation unless a certain caveat was fulfilled (for example: If the participants had known R’s wishes) | 62 (7.8) |
| Not comfortable to assent to R’s donation as they did not fully understand organ donation | 59 (7.4) |

**Supplementary Table 4C: Suggestion by participants on measures that can be put in place to increase acceptance of organ donation (Question 36)**

| **Explanation** | **N (%)** |
| --- | --- |
| Education: Greater awareness and discussion on HOTA | 320 (40.1) |
| Education: Knowing more about the process of organ harvesting | 119 (14.9) |
| Education: Being more aware of the beneficial impacts | 133 (16.7) |
| Education: Educate older generation/target elderly | 3 (0.4) |
| Protocols taken to ensure safe and ethical organ harvesting e.g. certification of brain-dead patients | 54 (6.8) |
| Earlier mental preparation from doctors on possible organ donation after death | 56 (7.0) |
| More time given to the family for grieving/acceptance of patient’s death before organ harvesting | 96 (12.0) |
| Better communication and empathy from doctors when discussing organ donation | 82 (10.3) |
| Knowing who the recipients of the organs are | 48 (6.0) |
| Organ recipients knowing who the donor is | 9 (1.1) |
| Getting to decide who the organs go to | 7 (0.9) |
| After second opinion is sought from another hospital | 7 (0.9) |
| Knowing the deceased's decisions/if donor had made his wishes known | 14 (1.8) |
| Allowing family members to have a say | 14 (1.8) |
| Compensation | 15 (1.9) |
| Modifications to the law | 17 (2.1) |
| Religious influence | 25 (3.1) |
| Replace with artificial organs so that body is still ‘whole’ | 1 (0.1) |
| Knowing health status of recipient/outcome of transplant | 1 (0.1) |

**Supplementary Table 5: Comparison of study participants to Singapore population distribution**

| **Demographic Characteristic** | **Study proportion (%)** | **Singapore population proportion (%)** |
| --- | --- | --- |
| *Age (years)*  21 – 40   1. – 60   > 60 | 29  42  29 | 24  40  24 |
| *Gender*  Male  Female | 42  58 | 51  49 |
| *Ethnicity*  Chinese  Malay  Indian  Others | 79  7  13  1 | 74  13  9  3 |
| *Religion*  Atheist  Buddhist  Christian  Muslim  Hindu  Taoist  Other | 24  25  32  9  7  3  0.5 | 18  33  19  14  5  11  - |
| *Marital status*  Single  Married  Divorced  Widowed | 24  71  2  2.5 | 31  60  4  5 |

**Supplementary Table 6: Data from other jurisditions and studies on survey and organ donation rates**

| Countries | Response Rates (%) | Survey results on willingness to be a donor (%) | Year of survey | Actual deceased donation rate (PMP) | Year of deceased donation rates | Population surveyed |
| --- | --- | --- | --- | --- | --- | --- |
| Armenia^1^ | 80 | 47.5 / 85.8 | 2001 | NA | N/A | Public / Physicians |
| Austria^2^ | NA | 39 | 2009 | 23.8 | 2019 | Public |
| Belgium^2^ | NA | 72 | 2009 | 30.3 | 2019 | Public |
| Chile^3^ | NA | 75 | 2011 | 10 | 2019 | Public |
| Colombia^4^ | NA | 81.8 | 2012 | 8.9 | 2019 | Public |
| Costa Rica^5^ | 22.3 | 84 / 75 | 2012 | 6.7 | 2019 | Physicians / Nurses |
| Croatia^2^ | NA | 53 | 2009 | 41.2 | 2019 | Public |
| Czech Republic^2^ | NA | 45 | 2009 | 25.51 | 2019 | Public |
| Ecuador^6^ (residing in Spain) | 94 | 59 | 2010 | 5 | 2019 | Public |
| Finland^2^ | NA | 71 | 2009 | 26.36 | 2019 | Public |
| France^2^ | NA | 66 | 2009 | 33.25 | 2019 | Public |
| Italy^2^ | NA | 49 | 2009 | 27.73 | 2019 | Public |
| Luxembourg^2^ | NA | 62 | 2009 | 15.80 | 2017 | Public |
| Norway^7^ | NA | 74 | 2015 | 18.78 | 2019 | Public |
| Poland^2^ | NA | 53 | 2009 | 12.96 | 2018 | Public |
| Russia^8^ | NA | 30 | 2017 | 3.9 | 2017 | Public |
| **Singapore**  ***(current study)*** | **35.3** | **85** | **2017** | **5.10** | **2010** | **Public** |
| Slovak Republic^2^ | NA | 48 | 2009 | 14.35 | 2018 | Public |
| Slovenia^9^ | 60 | 65 | 2017 | 24.67 | 2018 | Public |
| Spain^10^ | NA | 68% | 2011 | 33.1 | 2019 | Public |
| Sweden^2^ | NA | 83% | 2009 | 19 | 2019 | Public |
| Tunisia^11^ | 100% | 50% | 2007 | 0.83 | 2013 | Public |
| Turkey^2^ | NA | 72.3 | 2009 | 7.47 | 2018 | Public |

1. Chekijian SA. *Legal, professional, public and policy barriers to the development of organ donation and transplantation programs in the Republic of Armenia*, Yale Medicine; 2001.

2. Communication DG. Organ donation and transplantation - Special Eurobarometer 333a. European Comission. 2010. Accessed 12th Feb 2020, Web site. <https://ec.europa.eu/commfrontoffice/publicopinion/archives/ebs/ebs_333a_en.pdf>.

3. Dominguez J. Presumed Consent Legislation Failed to Improve Organ Donation in Chile. *Transplantation Proceedings.* 2013;45:Pages 1316-131. <https://doi.org/10.1016/j.transproceed.2013.01.008>.

4. David Andrés Castañeda-Millán MD FA, Diego Ovalle, Claudia Martínez. Attitudes and beliefs about organ donation in Colombia: Where should efforts be focused to improve national donation rates? *Journal of the Faculty of Medicine.* 2013;62. <http://dx.doi.org/10.15446/revfacmed.v62n1.43660>.

5. G. Söffker MB, T. Welte ,M. Quintel &S. Kluge Recruitment of intensive care specialists for postmortem organ donation in Germany. *Med Klin Intensivmed Notfmed* 2014;109:41-47. <https://doi-org.libproxy1.nus.edu.sg/10.1007/s00063-013-0271-x>.

6. Ríos A1 L-NA, Navalón JC, Martínez-Alarcón L, Ayala-García MA, Sebastián-Ruiz MJ, Moya-Faz F, Garrido G, Ramirez P, Parrilla P. The Latin American population in Spain and organ donation. Attitude toward deceased organ donation and organ donation rates. *Transplantation International.* 2015. <https://www-ncbi-nlm-nih-gov.libproxy1.nus.edu.sg/pubmed/25557362>.

7. Stiftelsen Organdonasjon. What do people in Norway think about donation and transplants? 2015. Accessed 12th Feb 2020, Web site. <https://organdonasjon.no/sporsmal/what-do-people-in-norway-think-about-donation-and-transplants/?lang=en>.

8. Stewart C. Willingness to donate organs in selected European countries 2018. statista. 2019. Accessed 12th Feb 2020, Web site. <https://www.statista.com/statistics/888928/attitudes-towards-organ-donation-in-europe-by-country/>.

9. Berzelak N, Avsec D, Kamin T. Reluctance and Willingness for Organ Donation After Death Among the Slovene General Population. *Zdr Varst.* 2019;58(4):155-163. doi:10.2478/sjph-2019-0020.

10. Scandroglio B, Dominguez-Gil B, Lopez JS, et al. Analysis of the attitudes and motivations of the Spanish population towards organ donation after death. *Transpl Int.* 2011;24(2):158-166. doi:10.1111/j.1432-2277.2010.01174.x.

11. Hamouda C1 BHM, Benzarti N, Zouari B. Tunisian survey about organ donation, attitude and opinion. *La Presse Médicale.* 2010;39. <https://www-sciencedirect-com.libproxy1.nus.edu.sg/science/article/pii/S0755498209003510?via%3Dihub>.
